# Heterogeneous network propagation with forward similarity integration to enhance drug–target association prediction

**DOI:** 10.7717/peerj-cs.1124

**Published:** 2022-10-11

**Authors:** Piyanut Tangmanussukum, Thitipong Kawichai, Apichat Suratanee, Kitiporn Plaimas

**Affiliations:** 1 Advanced Virtual and Intelligent Computing (AVIC) Center, Department of Mathematics and Computer Science, Faculty of Science, Chulalongkorn University, Bangkok, Thailand; 2 Department of Mathematics and Computer Science, Academic Division, Chulachomklao Royal Military Academy, Nakhon Nayok, Thailand; 3 Department of Mathematics, Faculty of Applied Science, King Mongkut’s University of Technology North Bangkok, Bangkok, Thailand; 4 Intelligent and Nonlinear Dynamics Innovations Research Center, Science and Technology Research Institute, King Mongkut’s University of Technology North Bangkok, Bangkok, Thailand; 5 Omics Science and Bioinformatics Center, Faculty of Science, Chulalongkorn University, Bangkok, Thailand

**Keywords:** Heterogeneous network, Network propagation, Similarity measures, Drug-target associations, Drug repurposing, Forward selection algorithm

## Abstract

Identification of drug–target interaction (DTI) is a crucial step to reduce time and cost in the drug discovery and development process. Since various biological data are publicly available, DTIs have been identified computationally. To predict DTIs, most existing methods focus on a single similarity measure of drugs and target proteins, whereas some recent methods integrate a particular set of drug and target similarity measures by a single integration function. Therefore, many DTIs are still missing. In this study, we propose heterogeneous network propagation with the forward similarity integration (FSI) algorithm, which systematically selects the optimal integration of multiple similarity measures of drugs and target proteins. Seven drug–drug and nine target–target similarity measures are applied with four distinct integration methods to finally create an optimal heterogeneous network model. Consequently, the optimal model uses the target similarity based on protein sequences and the fused drug similarity, which combines the similarity measures based on chemical structures, the Jaccard scores of drug–disease associations, and the cosine scores of drug–drug interactions. With an accuracy of 99.8%, this model significantly outperforms others that utilize different similarity measures of drugs and target proteins. In addition, the validation of the DTI predictions of this model demonstrates the ability of our method to discover missing potential DTIs.

## Introduction

The identification of drug targets is a very important part of drug development. Not only does it help to gain a better understanding of drug mechanisms and side effects, but it also provides opportunities to enhance drug selectivity and enable drug repurposing (Butina, Segall & Frankcombe, 2002; Schenone et al., 2013). There are several wet-lab techniques for identifying drug targets, such as biochemical affinity purification and genetic modifications (Schenone et al., 2013). However, only a limited number of drug targets were experimentally discovered and validated due to the high costs and time involved in conducting the experimental labs (Rudrapal, Khairnar & Jadhav, 2020).

To alleviate the bottleneck problem of drug target identification, computational inference methods were employed to discover potential drug–target interactions (DTIs). Existing computational methods can be broadly assorted into three categories: ligand-based, docking simulation, and chemogenomic methods (Bhargava, Sharma & Suravajhala, 2021). The ligand-based methods use similarities between ligands to infer potential DTIs (Shaikh et al., 2021; Yang et al., 2021). For docking simulation methods, three-dimensional (3D) structures of target proteins are usually required to simulate molecular docking (Al-Karmalawy et al., 2021; Alonso, Bliznyuk & Gready, 2006; Cheng et al., 2007; Pinzi & Rastelli, 2019). Nevertheless, the limited availability of 3D structural data of target proteins is a big obstruction to these methods. In chemogenomic methods, various properties of drugs and targets are used to predict DTIs via machine learning models and network-based models.

Most of the existing chemogenomic methods are based on the similarity-based technique, which infers promising DTIs based on drug–drug and target–target similarities. Typically, chemical structures of drugs and protein sequences of target proteins are used to formulate drug–drug and target–target similarity measures, respectively. For example, Bleakley & Yamanishi (2009) developed a bipartite local model (BLM) using similarities based on chemical and genomic data for predicting unknown DTIs. Wang, Yang & Li, (2013a) proposed heterogeneous graph-based inference (HGBI), which uses similarity scores based on drug chemical structures and target protein sequences to form a heterogeneous network and predict new drug–target links by using an information propagation algorithm. Zhang et al. (2022) proposed an unsupervised clustering model for DTI predictions based on structural similarity of drugs and protein sequences which combines OPTICS and BGMM algorithms using to detect noise and extract significant interaction pairs.

**Figure 1:**
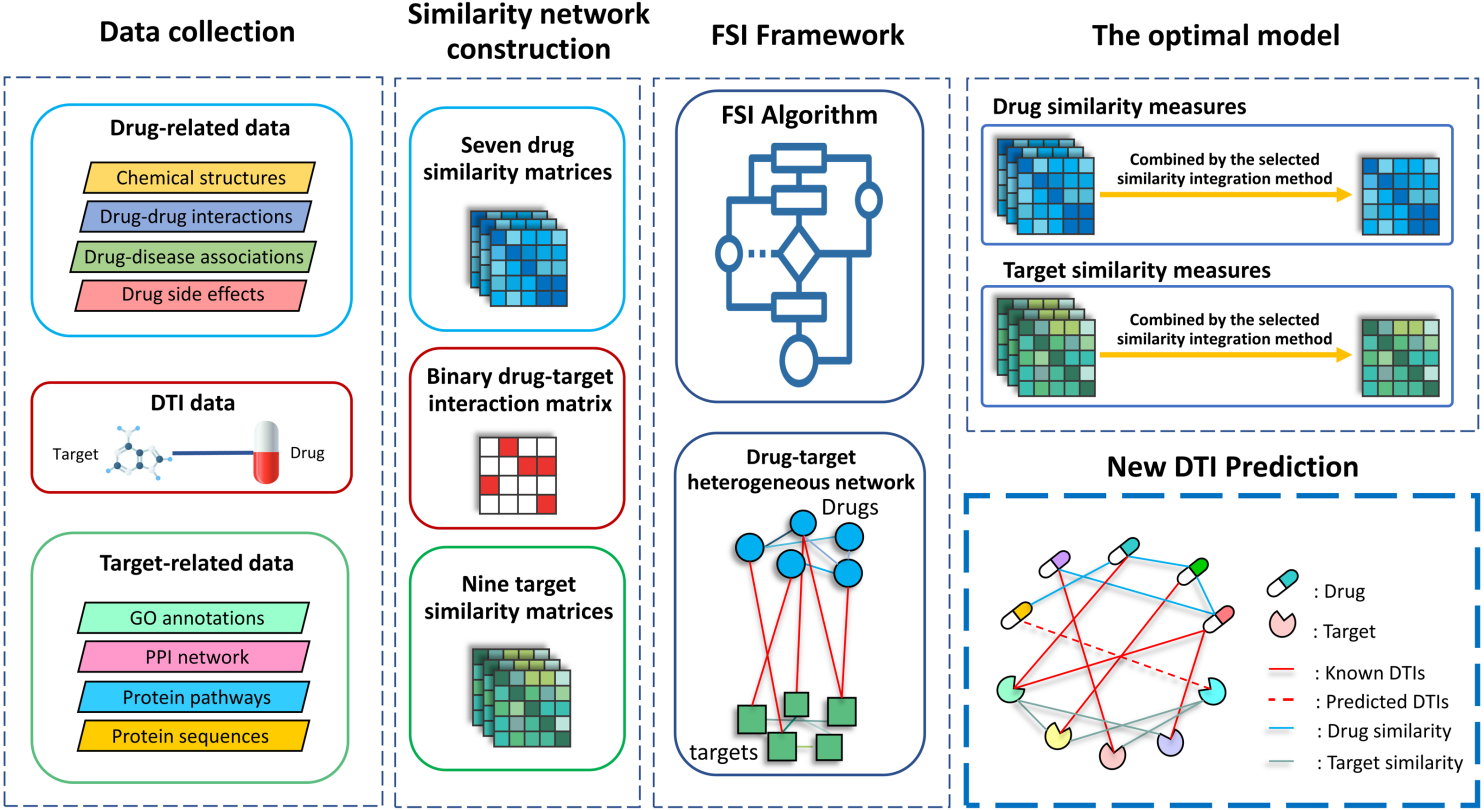
An overview of this study. First, structural data, molecular interaction, and phenotypic data of drugs were collected to generate seven drug–drug similarity measures. Second, genomic data, molecular interaction, and functional data of drug target proteins were used to create nine target–target similarity measures. Third, various integration functions, including both linear integration functions (*i.e.*, average, maximum, and minimum) and non-linear integration functions (*i.e.*, SNF) were applied to combine multiple similarity measures. Then, a heterogeneous network model with an optimal similarity integration was created along with the FSI algorithm framework. The obtained optimal model was then used to predict DTIs and measured the performance of the network propagation model. Finally, our predictions were validated by searching for literature support.

With the rapid growth of high-throughput experiments and information technology, extensive biological data are available in public databases. Many recent methods have created integrated drug–drug and target–target similarities based on various drug-related and target-related data to enhance the performance of models to predict DTIs. In addition, several techniques were used to integrate the multiple domains of these data, such as linear functions (*e.g.*, average, maximum, *etc.*) (Cheng et al., 2013; Kim, A-s & Nam, 2019; Thafar et al., 2020), the similarity network fusion (SNF) (Rohani & Eslahchi, 2019; Thafar et al., 2020), and low-dimensional feature learning (*e.g.*, random walk algorithms) (An & Yu, 2021; Luo et al., 2017). However, most of these methods integrate all data regardless of optimal dataset selections. In addition, they used only a single integration function without comparing distinct integration functions to identify an optimal function that fits the data. These probably lead to the non-optimal performance of predictions. Moreover, it has been known that network-based methods have been successfully applied in several applications (Hengphasatporn et al., 2020; Janyasupab, Suratanee & Plaimas, 2021; Kawichai, Suratanee & Plaimas, 2021; Suratanee, Buaboocha & Plaimas, 2021; Suratanee et al., 2018; Suratanee & Plaimas, 2014; Suratanee & Plaimas, 2015; Suratanee & Plaimas, 2017; Suratanee & Plaimas, 2018; Suratanee & Plaimas, 2020; Suratanee & Plaimas, 2021). Therefore, in this work, we propose a network-based method with the forward similarity integration (FSI) framework to systemically integrate multiple similarities and predict new links between drugs and targets. A schematic diagram illustrating an overview of this work is shown in Fig. 1. Structural data, molecular interaction and phenotypic data of drugs were collected to generate seven drug–drug similarity measures. Genomic data, molecular interaction and functional data of drug target proteins were used to create nine target–target similarity measures. To combine multiple similarity measures, we considered various integration functions, including both linear integration functions (*i.e.*, average, maximum, and minimum) and non-linear integration function (*i.e.*, SNF). The network-based method with the FSI framework was designed to systematically create a heterogeneous network model with an optimal similarity integration. To predict the links of DTIs, we used the best model constructed by the FSI algorithm and performed network propagation. Finally, we validated our link predictions by searching for literature support. The main contribution of this research is to improve the performance of a heterogeneous network model to predict DTIs. The proposed method is a well-suited algorithm to automatically select the best set of similarities and assign suitable weights for network propagation to yield better scores for unknown DTIs. At present, most of existing methods are based on machine learning with various features involved. However, the principle of the heterogeneous network model is a suitable way to directly propagate the links between drugs and targets. It is also an easy task which can be run faster than the machine learning approaches. Therefore, our proposed algorithm is useful and can be used to improve the heterogeneous network model.

## Material and Methods

### Data collection

First, we downloaded all known DTIs from DrugBank (Wishart et al., 2018). For each drug and target, various aspects of drug and target data from multiple levels of the biological system, including chemical, molecular, phenotypic, and functional data, were collected. The drug and target protein data were accumulated from several public databases, as shown in Table S1.

For the drug data, we downloaded chemical structures and a list of drug–drug interactions—one drug affects the molecular activity of another—from DrugBank (Wishart et al., 2018). Drug–disease association data, in which drugs exert effects on diseases, were collected from the Comparative Toxicogenomics Database (CTD) (Davis et al., 2021). Moreover, we downloaded the data on drug side effects (SEs)—undesirable effects that occur in addition to therapeutic effects—from SIDER (Kuhn et al., 2016). Both drug-disease associations (DDAs) and SEs can represent the phenotypic information of drugs.

In terms of drug target proteins, we collected protein sequences from DrugBank (Wishart et al., 2018). We also used the protein–protein interaction (PPI) network, downloaded from STRING (Szklarczyk et al., 2021), as the molecular interaction data of target proteins. We used gene ontology (GO) annotation data from Gene Ontology Annotation (GOA) (Huntley et al., 2009) and used them as the functional information of target proteins. Pathway information related to target proteins was accumulated from the Kyoto Encyclopedia of Genes and Genomes (KEGG) (Kanehisa & Goto, 2000). Only drugs and target proteins available in all distinct datasets were used. Finally, we obtained 862 drugs, 1,517 target proteins and 3,583 known DTIs.

### Drug–drug and target–target similarity measures

Based on four data sets of drugs and four data sets of target proteins, we can formulate seven drug–drug similarity measures and nine target–target similarity measures. The seven drug–drug similarity measures and their defined abbreviations are shown in Table S2 . Similarity scores between the two drugs based on their chemical structures (Structures), explained in a simplified molecular-input line-entry system (SMILES) (Weininger, 1988), are computed by the Chemical Development Kit (CDK) (Davis et al., 2021). We used the Tanimoto score (Tanimoto, 1958) to measure structural similarity between two drugs following Eq. (1), where *r*_*i*_ and *r*_*j*_ are binary vectors of the fingerprints of drugs and $ \left\| \cdot \right\| $ represents the length of a binary vector.

(1) \begin{eqnarray*}{S}_{Tanimoto} \left( {r}_{i},{r}_{j} \right) = \frac{{r}_{i}\cdot {r}_{j}}{{ \left\| {r}_{i} \right\| }^{2}+{ \left\| {r}_{i} \right\| }^{2}-{r}_{i}\cdot {r}_{j}} .\end{eqnarray*}

To measure drug–drug similarity based on DDAs, drug–drug interactions (DDIs), and SEs, we used two well-known similarity indices: the Jaccard index (Jaccard, 1912) and the cosine index (Singhal, 2001). Both similarity indices are frequently applied to measure drug–drug similarity in numerous studies (Gottlieb et al., 2011; Öztürk, Ozkirimli & Özgür, 2016; Perlman et al., 2011; Zhao et al., 2022) due to their simple calculations and similarity measuring efficiencies. With these indices, we obtained six different drug–drug similarity measures based on DDA, DDI, and SE, as shown in Table S2. The Jaccard and cosine similarity indices are defined in Eqs. (2) and (3), where *u* and *v* are binary vectors and $ \left\| \cdot \right\| $ represents the length of a binary vector. For each drug, a value of one in a binary vector represents an existing association with a disease, an existing interaction with another drug, and an existing SE.

(2) \begin{eqnarray*}& & {S}_{Jaccard} \left( u,v \right) = \frac{u\cdot v}{\parallel u{\parallel }^{2}+\parallel v{\parallel }^{2}-u\cdot v} \end{eqnarray*}

(3) \begin{eqnarray*}& & {S}_{Cosine} \left( u,v \right) = \frac{u\cdot v}{\parallel u\parallel \cdot \parallel v\parallel } .\end{eqnarray*}

For target–target similarity measures, we used four data sets of drug target proteins to obtain nine target–target similarity measures. The methods applied to measure the target–target similarities and their defined abbreviations are summarized in Table S3.

Based on the protein sequences of drug targets, we applied the Smith–Waterman algorithm (Smith & Waterman, 1981) and the Needleman–Wunsch algorithm (Needleman & Wunsch, 1970) to produce the scores of local sequence alignments (Seq_Loc) and global sequence alignments (Seq_Glo), respectively. Then, the similarity scores were normalized from 0 to 1 using the formula suggested by Wang, Yang & Li (2013b).

In the STRING (Szklarczyk et al., 2021) database, each protein pair in the PPI network is explained with a confidence score that indicates how much the interaction between two proteins is more likely to be true (Yu & Finley Jr, 2009). To reconstruct a PPI network with more reliable interactions, we kept only the PPIs whose confidence scores were greater than or equal to 0.5. To measure the similarity between the two target proteins, we applied Dijkstra’s algorithm to find the distances of the shortest paths linking the two protein nodes in the PPI network. Then, the distances were transformed to the inverse shortest path similarity (PPI_ISP) scores in Eq. (4), where *S*(*p*, *p*′) is the PPI_ISP similarity score between two proteins and *D*(*p*, *p*′) is the distance of the shortest path linking between those proteins in the PPI network. According to Perlman et al. (2011), parameters A and b were chosen to be 0.9 and 1, respectively.

(4) \begin{eqnarray*}S \left( p,{p}^{{}^{{^{\prime}}}} \right) =A{e}^{-bD(p,{p}^{{^{\prime}}})}.\end{eqnarray*}

In addition to PPI_ISP, we also measured the similarity between two target proteins based on their common neighbors in the PPI network by using the Jaccard index and cosine index, denoted as PPI_Jac and PPI_Cos, respectively.

Based on GO annotations of target proteins, we used GOSemSim (Yu, 2020) in the R package to compute the semantic similarity scores between the two target proteins. The methods used for semantic measurement were the Wang (Yu et al., 2010) and Jiang (Yu et al., 2010) methods. With these two methods, we denoted the target–target similarity scores based on the GO annotations as GO_Wang and GO_Jiang, respectively. Furthermore, we computed the target–target similarity scores based on the list of pathways in which each target protein is involved by using the Jaccard and cosine indices, following Eqs. (2) and (3). According to these two indices, we denoted the similarity measures by PW_Jac and PW_Cos, respectively.

### Heterogeneous network propagation

**Figure 2:**
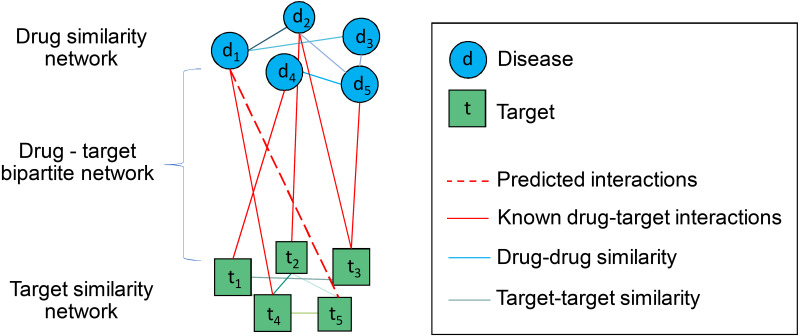
Structure of the drug–target heterogeneous network.

The structure of the drug–target heterogeneous network in this work is illustrated in Fig. 2. This heterogeneous network is composed of three compartmentalized networks: a drug similarity network, a target similarity network, and a drug–target bipartite network.

In the drug similarity network, we used *D* = *d*_1_, *d*_2_, *d*_3_, …, *d*_*m*_ to denote the set of *m* drug nodes and *E*_*dd*_to denote the set of edges linking between any two drug nodes. Based on a particular drug–drug similarity measure, the similarity scores between two drugs were calculated to represent the edge’s weights *W*_*dd*_. Similarly, let *T* = *t*_1_, *t*_2_, *t*_3_, …, *t*_*n*_ denote the set of *n* target protein nodes and *E*_*tt*_ denote the set of edges in the target similarity network. Based on a defined target–target similarity measure, *W*_*tt*_ contains the similarity scores that serve as the edge weights of the target similarity network. In the drug–target bipartite network, let *E*_*dt*_ be the set of edges linking between drug and target nodes; *W*_*dt*_ denotes by the weights of the edges of the drug–target bipartite network. Each value in *W*_*dt*_ can be either one or zero, where one represents the presence of an edge or a DTI and zero represents the absence of an edge or a DTI. The drug–target heterogeneous network (*G*_*DT*_) can be formulated as shown in Eq. (5).

(5) \begin{eqnarray*}{G}_{DT}= \left\{ D,T \right\} , \left\{ {E}_{dd},{E}_{tt},{E}_{dt} \right\} ,{W}_{dd},{W}_{tt},{W}_{dt}.\end{eqnarray*}

The *G*_*DT*_ network is an incomplete graph where there are some missing edges between the drug and target nodes. To infer these missing edges, we employed the heterogeneous network propagation algorithm, which was broadly utilized in numerous applications, such as the identification of DTIs (Peng, Li & Shang, 2020; Rayhan et al., 2020), the prediction of DDAs (Fiscon & Paci, 2021; He, Yang & Gong, 2020), and other applications (Eswaran et al., 2017; Yang, Zhang & Han, 2019; Yang et al., 2018). In this algorithm, the weights of all networks are considered matrices (*i.e.*, *W*_*dd*_ ∈ ℝ^*m*×*m*^, *W*_*tt*_ ∈ ℝ^*n*×*n*^, and *W*_*dt*_ ∈ ℝ^*m*×*n*^), where *m* and *n* are the numbers of drugs and target proteins, respectively. This algorithm iteratively updates the weights in *W*_*dt*_ by propagating the weights in the drug similarity network, drug–target network, and target similarity network, as shown in Eq. (6).

(6) \begin{eqnarray*}{W}_{dt}^{i+1}=\alpha {W}_{dd}\times {W}_{dt}^{i}\times {W}_{tt}+ \left( 1-\alpha \right) {W}_{dt}^{0}.\end{eqnarray*}

In this formula, ${W}_{dt}^{0}$ is the matrix of the initial weights of the edges linking drugs and targets, which can be either one or zero following the list of known DTIs. *α* is a decay factor, that is in the range of 0 to 1. This parameter indicates how much the propagation of the networks’ weights affects the newly updated *W*_*dt*_ when compared to the effects of ${W}_{dt}^{0}$. The formulation in Eq. (6) will converge if the *W*_*dd*_ and *W*_*tt*_ are properly normalized, as shown in Eqs. (7) and (8) (Wang, Yang & Li, 2013b).

(7) \begin{eqnarray*}w \left( {d}_{i},{d}_{j} \right) & = \frac{w \left( {d}_{i},{d}_{j} \right) }{\sqrt{\sum _{k=1}^{m}w \left( {d}_{i},{d}_{k} \right) \sum _{k=1}^{m}w \left( {d}_{k},{d}_{j} \right) }} \end{eqnarray*}

(8) \begin{eqnarray*}w \left( {t}_{i},{t}_{j} \right) & = \frac{w \left( {t}_{i},{t}_{j} \right) }{\sqrt{\sum _{k=1}^{n}w \left( {t}_{i},{t}_{k} \right) \sum _{k=1}^{n}w \left( {t}_{k},{t}_{j} \right) }} .\end{eqnarray*}

### Forward similarity integration (FSI)

In the drug–target heterogeneous network, we are interested in integrating multiple similarity measures on the drug and target similarity networks to enhance the performance in predicting DTIs. We propose the FSI algorithm for systematically selecting and combining multiple similarity measures. The implementation of this FSI algorithm and the data table of drug-drug, target-target similarities are provided at https://github.com/piyanuttnk/FSI_framework.

Suppose that there are *k* different drug–drug similarity measures in set *AD* = {*dd*_1_, *dd*_2_, …, *dd*_*k*_} and *l* different target–target similarity measures in set *AT* = {*tt*_1_, *tt*_2_, …, *tt*_*l*_}. To create an integrated similarity measure by function *IntegrateSim* based on a particular performance measure, the FSI algorithm finds the optimal subsets of drug–drug and target–target similarity measures stepwise, denoted as *OD* and *OT*, respectively. The pseudocodes explaining the FSI algorithm are shown in Algorithm 1.

In each step, a drug–drug and a target–target similarity measures are added into sets *OD* and *OT*, respectively, to form different heterogeneous network models. These models were compared with their performance (*PRF*) to select the best one, which was added to sets *OD* and *OT*. Then, the drug–drug and target–target similarity measures recently added to the optimal subsets are removed from the remaining sets of drug–drug and target–target similarity measures, denoted as *RD* and *RT*, respectively. The FSI algorithm iteratively updates *OD* and *OT* by adding a drug–drug and target–target similarity measure until the performance improved no more from the addition, or there were no similarity measures remaining in *RD* or *RT*. Therefore, we anticipate that a heterogeneous network model formed with the integrated similarity measures obtained by the FSI algorithm will show the best performance in predicting DTIs. However, the optimal subsets of similarity measures obtained by the FSI (*OD* and *OT*) may depend on the performance measure used to evaluate the heterogeneous network models and the integration method applied. In this study, we considered several performance measures used in the FSI and several integration functions to create an integrated similarity matrix from multiple similarity measures.

**Algorithm 1:** Forward Similarity Integration (FSI)

Input: A set of all drug-drug similarity measures (*AD*) and target-target similarity measures (*AT*)

Output: An optimal subset of drug-drug similarity measures (*OD*) and target-target similarity measures (*OT*), which are orderly integrated to obtain the most optimally combined drug-drug and target-target similarity measures

1. **Initialize** *k* = 0, *OD*_0_, *OT*_0_ = ∅, *RD*_0_ = *AD*, *RT*_0_ = *AT*, *PRF*_0_ = 0.
2. **repeat**
3. *k* = *k* + 1
4. x*, y* = **argmax**_*x*∈*RD*__k−1__,*y*∈*RT*__k−1_ [EstimatePRF(IntegrateSim(*OD*_*k*__−1_ ∪ {*x* }), IntegrateSim(*OT*_*k*__−1_ ∪ {*y* })))] // Add both drug and target similarity
5. **Denote** the best performance as *PRF*_*both*_, X* = {x* }, and Y* = {y* }
6. **if** *k* == 1 **then**
7. *PRF*_*k*_ = *PRF*_*both*_
8. **else** // Also consider adding only drug or target similarity
9. *x*_d_* = **argmax**_*x*∈*RD*__k−1_ [EstimatePRF(IntegrateSim(*OD*_*k*__−1_ ∪ {*x* }), IntegrateSim(*OT*_*k*__−1_)))] **and denote** the best performance as *PRF*_*drug*_
10. *y*_t_* = **argmax**_*y*∈*RT*__k−1_ [EstimatePRF(IntegrateSim(*OD*_*k*__−1_), IntegrateSim(*OT*_*k*__−1_ ∪ {*y* })))] **and denote** the best performance as *PRF*_*target*_
11. *PRF*_*k*_ = **max** (*PRF*_*both*_, *PRF*_*drug*_, *PRF*_*target*_)
12. **if** *PRF*_*k*_ == *PRF*_*drug*_ **then**
13. X* = {*x*_d_* } and Y* = ∅
14. **else if** *PRF*_*k*_ == *PRF*_*target*_ **then**
15. X* = ∅ **and** Y* = {*y*_t_* }
16. **end if**
17. **end if**
18. **if** *PRF*_*k*_ >*PRF*_*k*__−1_ **then**
19. **Update** *OD*_*k*_ *= OD*_*k*__−1_ ∪ X*, OT_*k*_ *= OT*_*k*__−1_ ∪ Y*, *RD*_*k*_ = *RD*_*k*__−1_ − X*, *RT*_*k*_ = *RT*_*k*__−1_ − Y*
20. **end if**
21. **until** (*PRF*_*k*_ ≤*PRF*_*k*__−1_ **or** —*RD*_*k*_— == 0 **or** —*RT*_*k*_— == 0)
22. return the lastest *OD* **and** *OT*

Based on a subset of similarity measures, we can create an integrated similarity matrix to form a heterogeneous network model following Algorithm 2. Let *SM* = {*s*_1_, *s*_2_, …, *s*_*p*_} be a set of given similarity measures that are arranged according to the order of integration, *Xs*_*i*_ be a matrix containing the similarity scores based on similarity measure *s*_*i*_, and *f* represents a function to combine two similarity matrices. The algorithm used to create an integrated similarity matrix (*IntSimMat*) based on a set of given similarity measures (*SM*) and an integration function *f*, is demonstrated in Algorithm 2, where $ \left\| \cdot \right\| $ is the number of elements in a set.

**Algorithm 2**: Integrating multiple similarity matrices

Input: A set of given similarity measures (*SM*) and a function of an integration method (*f*)

Output: An integrated similarity matrix (*IntSimMat*)

1. **for** *i* = 1 **to** *i* = —*SM*—− 1
2. **if** *i* == 1 **then**
3. *IntSimMat* = *f* (*Xs*_*i*_, *Xs*_*i*__+1_)
4. **else**
5. *IntSimMat* = *f* (*IntSimMat*, *Xs*_*i*__+1_)
6. **end if**
7. end for

According to Algorithm 2, *Xs*_*i*_ can be either a drug–drug similarity matrix (*i.e.*, *Xs*_*i*_ ∈ ℝ^*m*×*m*^) or a target–target similarity matrix (*i.e.*, *Xs*_*i*_ ∈ ℝ^*n*×*n*^). In this study, we included both linear and non-linear functions for integrating multiple similarity matrices: functions average (*AVG*), maximum (*MAX*), minimum (*MIN*), and *SNF* (Wang et al., 2014). *AVG*, *MAX*, and *MIN* are defined in Eqs. (9) to (11), where *A* = [*a*_*kl*_] and *B* = [*b*_*kl*_] are two different matrices of similarity scores.

(9) \begin{eqnarray*}AVG \left( A,B \right) & = \left[ \frac{{a}_{kl}+{b}_{kl}}{2} \right] \end{eqnarray*}

(10) \begin{eqnarray*}MIN \left( A,B \right) & = \left[ min \left( {a}_{kl},{b}_{kl} \right) \right] \end{eqnarray*}

(11) \begin{eqnarray*}MAX \left( A,B \right) & = \left[ max \left( {a}_{kl},{b}_{kl} \right) \right] .\end{eqnarray*}

SNF (Wang et al., 2014) is a method that calculates and fuses similarity networks into a single similarity network. The SNF utilizes an iterative non-linear approach and updates the global similarity network of each layer using a local *k*-nearest neighbors (*k* NN) approach. A similarity value between nodes is propagated to the *k* NN.

In formal, ${W}^{ \left( m \right) }$ is denoted as the similarity matrix for the *m*th data type. Initially, a transition probability matrix between all nodes is defined in Eq. (12), and a transition probability matrix between nearest neighbors is defined in Eq. (13), where *N*_*i*_ is a set of node *i*’s *k* NN in ${W}^{ \left( m \right) }$ matrices.

(12) \begin{eqnarray*}{P}_{0}^{ \left( m \right) } \left( i,j \right) = \left\{ \begin{array}{@{}l@{}} \displaystyle \frac{{W}^{ \left( m \right) } \left( i,j \right) }{2\sum _{k\not = i}{W}^{ \left( m \right) } \left( i,k \right) } ,j\not = i; \\ \displaystyle \frac{1,j=i.}{2} , \end{array} \right. \end{eqnarray*}

(13) \begin{eqnarray*}{S}^{ \left( m \right) } \left( i,j \right) = \left\{ \begin{array}{@{}l@{}} \displaystyle \frac{{W}^{ \left( m \right) }(i,j)}{\sum _{k\in {N}_{i}}{W}^{ \left( m \right) }(i,k)} ,j\in {N}_{i}; \\ \displaystyle 0,\text{Otherwise}. \end{array} \right. \end{eqnarray*}

*P* is iteratively updated using the values passing between the nearest neighbors following Eq. (14), where ${P}_{q}^{ \left( m \right) }$ is the matrix for *m*th data type at iteration *q* and *M* is the number of data types. Finally, the overall status matrix at iteration *t*^*th*^ is calculated, as shown in Eq. (15).

(14) \begin{eqnarray*}{P}_{q+1}^{ \left( m \right) }& ={S}^{ \left( m \right) } \frac{\sum _{k\not = m}{P}_{q}^{ \left( m \right) }}{M-1} {S}^{{ \left( m \right) }^{q}}\end{eqnarray*}

(15) \begin{eqnarray*}{P}^{ \left( t \right) }& = \frac{\sum _{m\in M}{P}_{q}^{ \left( m \right) }}{M} .\end{eqnarray*}

### Experimental environments

Our experiments can be divided into four parts. The first part is an identification of the optimal decay factor to reduce the model variable. The second part is a selection of the heterogeneous network model to find the optimal model by the FSI. The third part is the performance comparison between the optimal model and other models, including full similarity integration, random similarity integrations, and the original heterogeneous network (OHN). The final part is an identification of new DTIs to predict new links between drugs and target proteins. All experiments were conducted on a 4.90-GHz core i7 laptop with 20 GB of ram under the environments of Python version 3.9. To estimate performance of each model, we used several packages comprising NumPy version 1.22.4, Pandas version 1.4.2, Scikit-learn version 1.1.2, Matplotlib version 3.5.2 and SNFpy version 0.2.2. To fairly compare the performance of different models, such as models with different values of the decay factors used or with different similarity integrations, training and testing data sets were controlled as the same.

### Performance evaluation

To evaluate the performance of all heterogeneous network models, we performed 10-fold cross-validation. All DTIs were randomly divided into 10 equal parts, where each part of positive and negative interactions was treated as test data in turn, and the remaining nine parts were used as training data. To completely evaluate the performance of all heterogeneous network models, we used general metrics, including the area under a precision–recall curve (AUPR), the area under a receiver operating characteristic curve (AUC), the F1-measure (F1), the precision (PRE), the recall (REC), the accuracy (ACC) and the Matthews correlation coefficient (MCC). These measures can be calculated as shown in Eqs. (16) to (20). TP, FP, FN, and TN denote the true positive, false positive, false negative, and true negative, respectively, which compares the predicted and actual classes.

(16) \begin{eqnarray*}PRE& = \frac{TP}{TP+FP} \end{eqnarray*}

(17) \begin{eqnarray*}REC& = \frac{TP}{TP+FN} \end{eqnarray*}

(18) \begin{eqnarray*}{F}_{1}measure& = \frac{2\cdot PRE\cdot REC}{PRE+REC} \end{eqnarray*}

(19) \begin{eqnarray*}ACC& = \frac{TP+TN}{TP+TN+FP+FN} \end{eqnarray*}

(20) \begin{eqnarray*}MCC& = \frac{TP\cdot TN-FP\cdot FN}{\sqrt{(TP+FP)\cdot (TP+FN)\cdot (TN+FP)\cdot (TN+FN)}} .\end{eqnarray*}

## Results

### Data summary

According to all the collected datasets of drugs and target proteins, we constructed a heterogeneous network with 862 drug nodes and 1,517 target nodes. All drug data (*i.e.*, chemical structures, DDIs, DDAs, and SEs) and all target data (*i.e.*, protein sequences, GO functions, PPI-based information, and pathway information) were collected. The network comprised 3,583 known DTIs linking drug and target nodes. This number of links is only 0.2740% of all possible edges linking drugs and targets. Thus, it is possible that many potential DTIs are still undiscovered in this dataset.

Figure S1 shows the degree distributions of the drugs and target proteins in the drug–target bipartite network, which can explain the overall picture of how many target proteins are associated with a drug and how many drugs are associated with a target protein according to the known DTIs. Notably, most drugs and target proteins are associated with a few target proteins and drugs, respectively. We found that 19.48% of the drugs and 38.85% of the target proteins had node degrees less than 5. The maximal degrees of the drug and target nodes were 61 and 40, respectively. A drug can bind to approximately four target proteins on average, and a target protein can interact with approximately two drugs on average. Additionally, the majority of nodes are rarely connected to one another while only a few nodes have dense links. This suggests that there would be many undiscovered links between drugs and target proteins in the drug–target bipartite network.

### Correlation analysis of similarity measures

**Figure 3:**
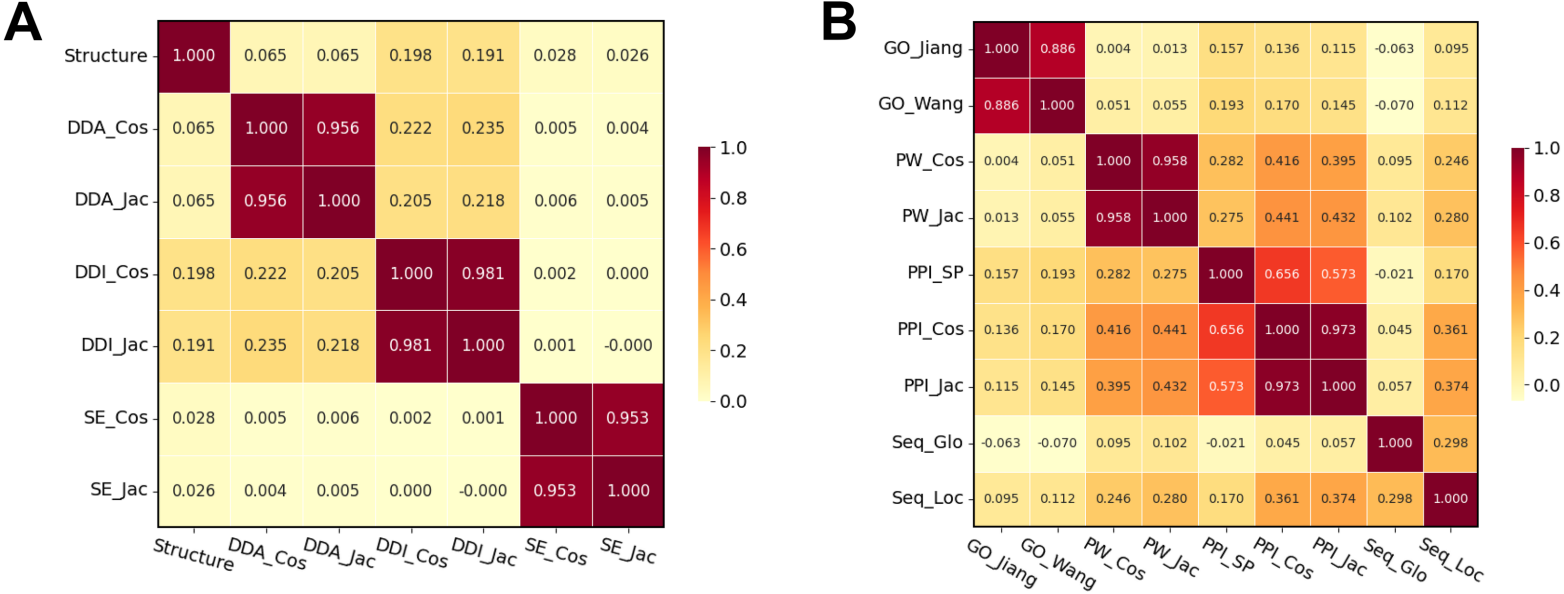
Heatmaps of the Pearson correlation coefficients of the drug and target similarity measures. (A) Drug similarity measures. (B) Target similarity measures.

In this section, we performed the Pearson correlation analysis between different drug–drug similarity measures and between different target–target similarity measures to preliminarily validate the orthogonality of the defined similarity measures. A Pearson correlation coefficient (*ρ*) is a measure of linear correlation between two data sets and assigns a value between −1 and 1, where 0 is no correlation, 1 is a total positive correlation, and −1 is a total negative correlation. The Pearson correlation coefficients that we calculate for all pairs of drug–drug and target–target similarity measures are shown in Fig. 3.

Usually, drug–drug similarity matrices and target–target similarity matrices are slightly positively correlated with one another. This suggests that drug and target similarity measures provide information that complements that of one another, which is useful when integrating them for predicting DTIs. However, the similarity measures based on the same datasets, such as DDI_Cos and DDI_Jac; PPI_ISP, PPI_Jac, and PPI_Cos, have their own highly positive correlation values. Thus, we avoid integrating drug or target similarity measures based on the same dataset in the FSI algorithm despite the different methods used to compute the similarity scores.

Interestingly, the drug similarity measures based on DDIs, chemical structures, and DDAs are negligibly positively correlated together (*ρ* ≤ 0.3). This suggests that the structural information of drugs is partly signified in DDIs and DDAs. Moreover, the target similarity measures based on PPI, protein sequences, and pathway information are slightly to moderately positively correlated together (*ρ* ≤ 0.5).

### Identification of the optimal decay factor

In the process of heterogeneous network propagation, one parameter required to be optimally tuned is the decay factor (*α*). This parameter indicates how much network propagation affects the weight updates of the edges linking the drug and the target nodes. Most studies (Luo et al., 2016; Yang et al., 2019) set the value of the decay factor at 0.4, which was suggested by Wang, Yang & Li (2013a). To discover the best model with an optimal similarity integration, we built numerous heterogeneous network models with different datasets combined and different integration methods used. With distinct data combinations, these models may have suitable values for the decay factor differently. To reduce the model variables, we preliminarily specified the value of the decay factor for all heterogeneous network models.

**Figure 4:**
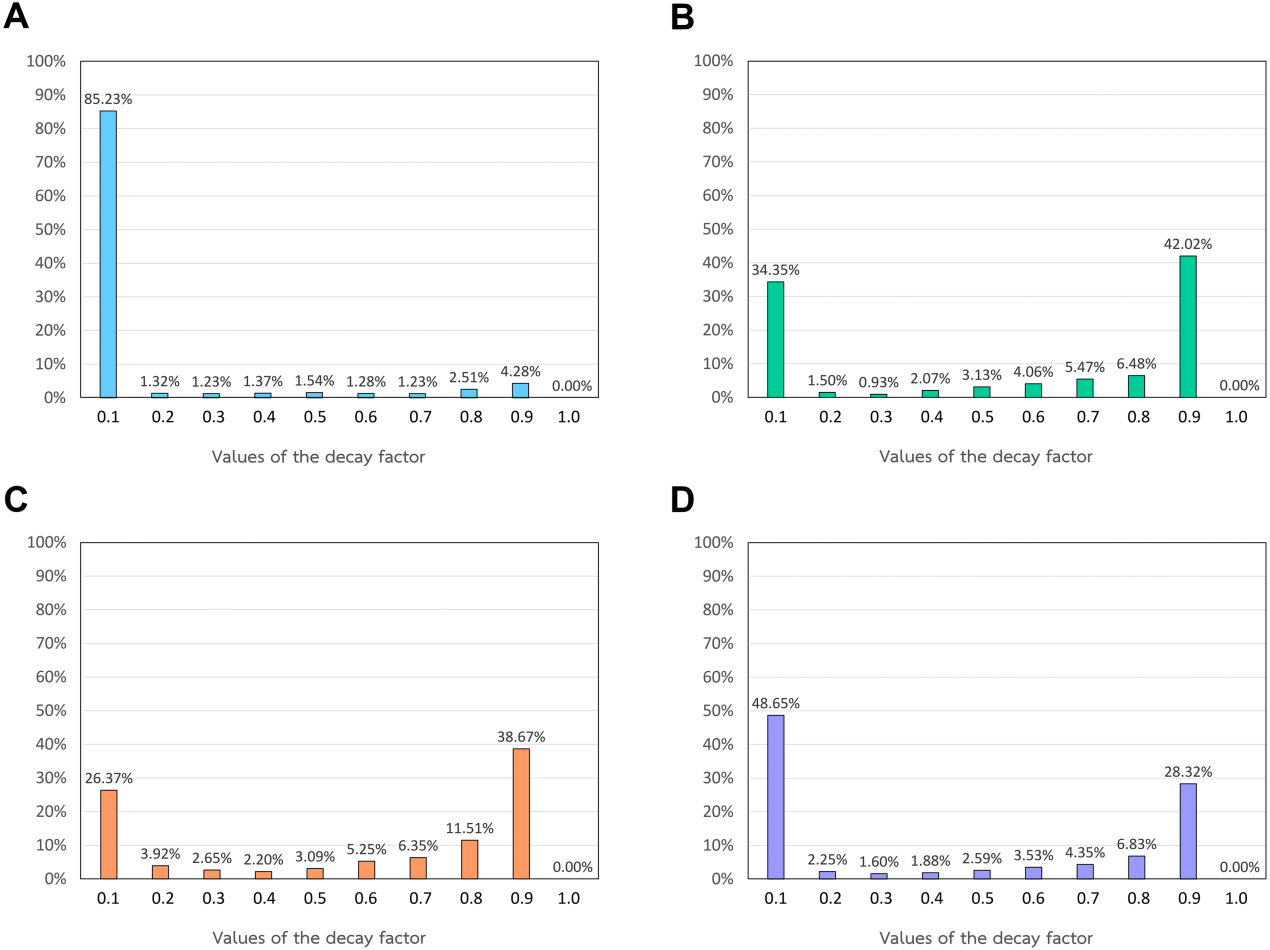
The percentage of models yielding high performances in different values of the decay factor. (A) The percentage of the models yielding the maximum AUC values. (B) The percentage of the models yielding the maximum AUPR values. (C) The percentage of the models yielding the maximum F1 values. (D) The percentage of the models yielding the maximum AUC/AUPR/F1 values (%).

Since there are seven drug–drug similarity measures and nine target–target similarity measures, we have 5,671 possible combinations of drug and target similarity measures to construct heterogeneous network models with different similarity integration. In this experiment, we randomly selected 10% of all possible combinations, resulting in 567 combinations of drug and target similarity measures. For each combination with multiple drugs and target similarity measures, we integrated those drug and target similarity measures by using all integration functions (*i.e.*, AVG, MAX, MIN, and SNF) to construct heterogeneous networks. In total, we have 567 different heterogeneous network models. In the network propagation of each model, we varied the values of the decay factor from 0.1 to 1, with a step of 0.1 and conducted 10-fold cross-validation to evaluate the model performance when using each value of the decay factor. For each model, we investigated the AUC, AUPR, and F1 values and used each of these metrics to select the optimal value of the decay factor. To compare all values of the decay factor, we counted the numbers of the models resulting in high performances in each decay factor value. The performances that we calculated were AUC, AUPR, and F1. The results are shown in Fig. 4.

Figure 4A shows that 85.23% of the models reach the maximum AUC values when setting the decay factor as 0.1. In Fig. 4B, 42.02% and 34.35% of the models yield maximum AUPR values when using decay factors of 0.9 and 0.1, respectively. Moreover, 38.67% and 26.37% of the models yields the maximum F1 scores when exploiting the decay factor as 0.9 and 0.1, respectively, as shown in Fig. 4C. To select the value of the decay factor that yields the best AUC, AUPR, and F1 values, we find the total number of models with the maximum AUC, AUPR, and F1 values as shown in Fig. 4D. As a result, it clearly shows that a decay factor of 0.1 yields the best coverage of the models with maximum AUC, AUPR, and F1 values. Therefore, we specified the value of the decay factor as 0.1 in the network propagation of all heterogeneous network models.

### Selection of the heterogeneous network model

Based on a given method of similarity integration, we introduce the FSI algorithm for discovering the best sets of drug and target similarity measures, which are used to combine and form a heterogeneous network model. Herein, we investigated three comprehensive evaluation metrics—AUC, AUPR, and F1—to serve as the criteria to select drug and target similarities to integrate into a heterogeneous network model. With the different similarity integration methods and evaluation metrics used in the FSI, we obtained eight different models as shown in Table 1.

**Table 1:** The FSI algorithm with different similarity integration methods and the performance measures used to select drug and target similarities.

**Similarity integration method**	**Performance measure for selecting drug/target similarities**		
	**AUC**	**AUPR**	**F1**
**AVG**	Model 1 {DDA_Jac}& {PPI_Jac, Seq_Loc}	Model 2 {DDA_Jac}&{Seq_Loc}	
**MAX**	Model 3 {DDA_Jac}& {PPI_Jac}		
**MIN**	Model 4 {DDA_Jac, Structures, DDA_Cos}&{PPI_Jac, Seq_Loc}	Model 5 {DDA_Jac, SE_Jac, Structures, DDI_Jac}&{Seq_Loc}	
**SNF**	Model 6 {DDA_Jac, DDI_Jac, PPI_Jac}&{Seq_Loc, PW_Jac}	Model 7 {DDA_Jac, DDI_Cos, Structures}&{Seq_Loc}	Model 8 {DDA_Jac, DDI_Jac, Structures}&{Seq_Loc}

As shown in Table 1, the FSI algorithm provides various output models generated by different integrations of drug and target similarities (either average (AVG), maximum (MAX), minimum (MIN), or similarity network fusion (SNF)) and different performance measures (AUC, AUPR, and F1 score). Each row in the table shows the best combination of similarities and integrations with the highest performance measures in column. For example, with the use of average (AVG) as a similarity integration method and the use of AUC as a performance evaluation, Model 1 using DDA_Jac, PPI_Jac for drug similarities, and Seq_Loc for target similarities yield the best AUC performance comparing to the other combinations of the similarity measures. The FSI presents the same or similar models when using AUPR or F1 as a performance evaluation for selecting drug and target similarities with either AVG, MAX, or MIN integration methods. We obtained the same model, formed by using DDA_Jac and Seq_Loc (Model 2) and the integration method AVG or MAX. FSI also returns Model 5 with using DDA_Jac, SE_Jac, Structures, and DDI_Jac for drug similarity and using Seq_Loc as a target similarity. Noticeably, DDA_Jac and Seq_Loc were always used in all obtained models because the models were preliminarily identified as the best by the FSI algorithm. Since this is an initial model where the FSI algorithm additionally selected other drug and target similarities into the heterogeneous model to increase the performance for DTIs predictions.

From these eight models in Table 1, we estimated the performance of each model by performing 10-fold cross-validation. The mean values of all evaluation metrics for each model are shown in Table 2. When we compared Model 1 and Model 2 by conducting *t*-tests, we found that Model 2 yielded significantly higher performances than Model 1 at a significance level of 0.05. Similar results were shown when we compared Model 1 and Model 3 or compared between Model 4 and Model 5. Moreover, the overall performance of Model 7 was significantly greater than that of Model 6 and 8 in most evaluation metrics, such as AUPR, F1, and MCC, at a significance level of 0.05. If we compared the models using the same integration methods, we found that using AUPR as a performance measure to select and integrate drug and target similarities to into a heterogeneous network model would produce a model with better performance than those using AUC or F1. Interestingly, Model 3 choose DDA_Jac and PPI_Jac as the optimal features when selecting the maximum as an integration method. Both similarities for drugs and targets are based on Jaccard indices. The maximum values of the overlap among the neighboring drugs and targets in the interaction network were much more important to yield better AUC. This might be because of the functional relation among drugs and their neighbors, as well as among targets and their similar neighboring targets. Model 4 provided DDA_Jac, Structures and DDA_Cos for drug similarities, and PPI_Jac and Seq_Loc for target similarities. Based on DDA_Jac and PPI_Jac in Model 4 with taking the maximum value, only one similarity was selected while in Model 3 with taking the minimum values, two or three similarities were selected. Therefore, taking the minimum value among the selected similarities could narrow the possibility of predicting an unrelated drug-target links with high score. In the better way, we should consider Structure and DDA_Cos also in the similarity integration for drugs and consider Seq_Loc more in the similarity integration for targets.

**Table 2:** Performance of eight FSI models and results of *t*-tests.

Model no.	AUPR	AUC	PRE	REC	F1	ACC	MCC
1	0.227	0.951	0.342	0.333	0.333	0.996	0.334
2	0.267^a,b^	0.935	0.389^a,b^	0.373^a,b^	0.379^a,b^	0.997^a,b^	0.378^a,b^
3	0.140	0.947	0.192	0.284	0.221	0.994	0.226
4	0.233	0.953	0.310	0.400	0.348	0.996	0.349
5	0.335^c^	0.926	0.426^c^	0.443^c^	0.434^c^	0.997^c^	0.433^c^
6	0.294	**0.958**	0.334	0.422	0.367	0.996	0.370
7	**0.481** ^d,e^	0.933^e^	**0.578** ^d^	0.508^d^	**0.539** ^d,e^	**0.998** ^d^	**0.540** ^d,e^
8	0.481	0.933	0.564	**0.515**	0.538	0.998	0.537

**Notes.**

The maximum value is shown in bold numbers.

a Significantly greater than the mean value of Model 1 at a significance level of 0.05.

b Significantly greater than the mean value of Model 3 at a significance level of 0.05.

c Significantly greater than the mean value of Model 4 at a significance level of 0.05.

d Significantly greater than the mean value of Model 6 at a significance level of 0.05.

e Significantly greater than the mean value of Model 8 at a significance level of 0.05.

According to the results in Table 2, it is noticeable that the maximum performance values are mostly of the models using SNF as a similarity integration method (Model 6, Model 7, and Model 8). Thus, we compared the performances of these models to select the best one. By performing *t*-tests, the mean values of almost all evaluation metrics, except that of AUC, of Model 7 are significantly greater than those of Model 6 at a significance level of 0.05. If we compare Model 7 and Model 8, the mean values of AUPR, AUC, F1, and MCC of Model 7 are significantly higher than those of Model 8 at a significance level of 0.05. Therefore, Model 7, which integrates DDA_Jac, DDI_Cos, and Structures into a drug similarity network by SNF and uses Seq_Loc as a target similarity, was selected as the best FSI model.

### Performance evaluation of an optimal model

**Figure 5:**
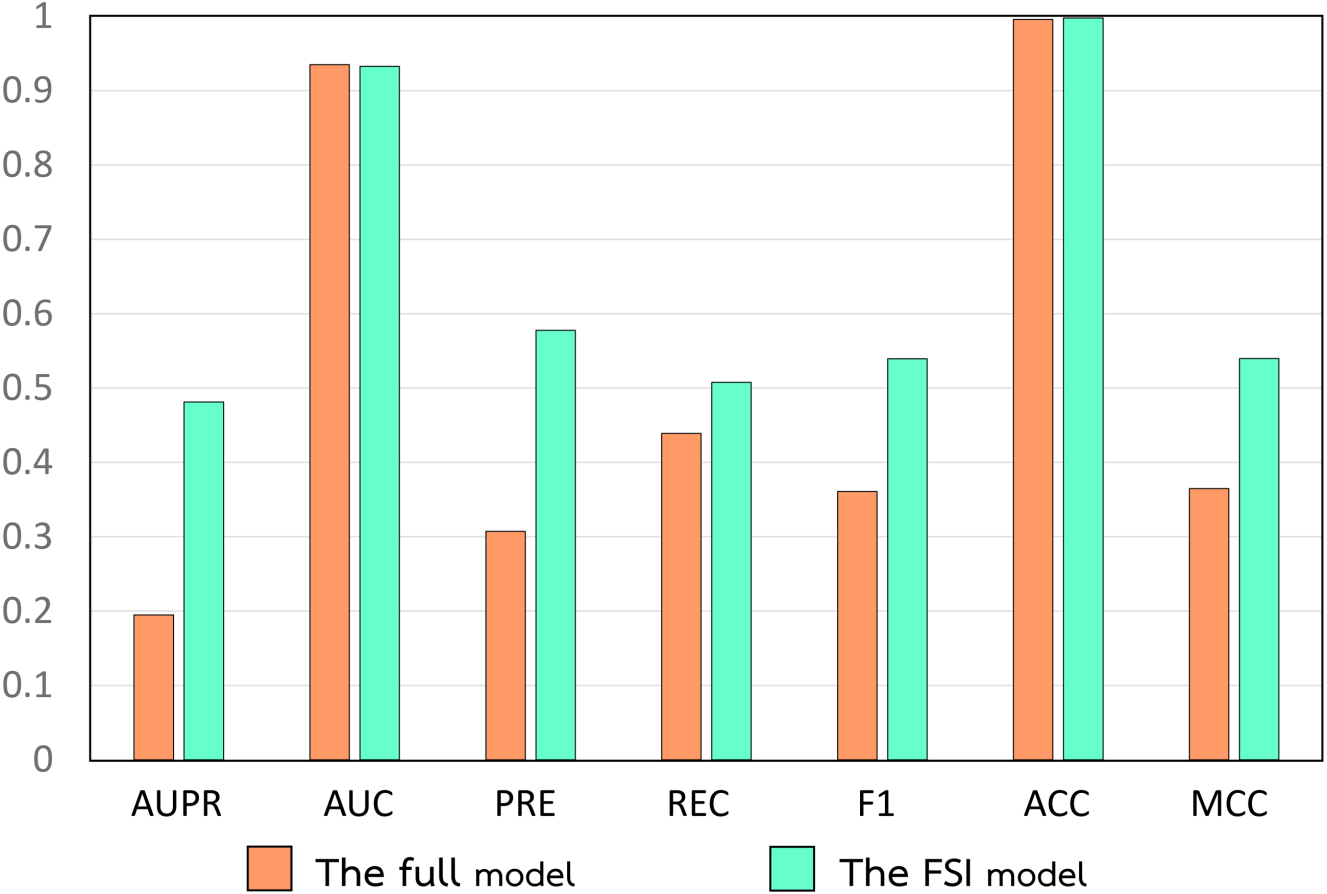
Performance comparison of the full model and the FSI.

In this section, we demonstrate the superior performance of the heterogeneous network model with the drug and target similarity integration selected by the FSI, termed the FSI model. This model combines DDA_Jac, DDI_Cos, and Structures as an integrated drug similarity using SNF and employs Seq_Loc as a target similarity. To verify that the model selected by FSI is the best, we compared its performance to those of the models with full similarity integration, random similarity integrations, and the original heterogeneous network (OHN) model, which uses only Structures and Seq_Loc, proposed by Liu et al. (2022), Liu et al. (2016), Peng et al. (2022) and Wang, Yang & Li (2013a). To demonstrate the efficiency of FSI, we first compare the performance of the FSI model with that of the full model which fuses all drug similarities and target similarities, as shown in Fig. 5.

As shown in Fig. 5, the FSI model performs much better than the full model. By *t*-tests, the mean values of all evaluation metrics of the FSI model, except AUC, were significantly greater than those of the full model at a significance level of 0.01, where *p*-values when considering AUPR, AUC, PRE, REC, F1, ACC, and MCC were 2.83E-13, 0.172, 3.10E-09, 2.79E-06, 1.99E-11, 1.81E-09, and 1.98E-11, respectively. This means that a model formed by selecting only some advantageous drug and target similarities by FSI is more efficient than a model that integrates all existing drug and target similarities without cautious consideration.

**Figure 6:**
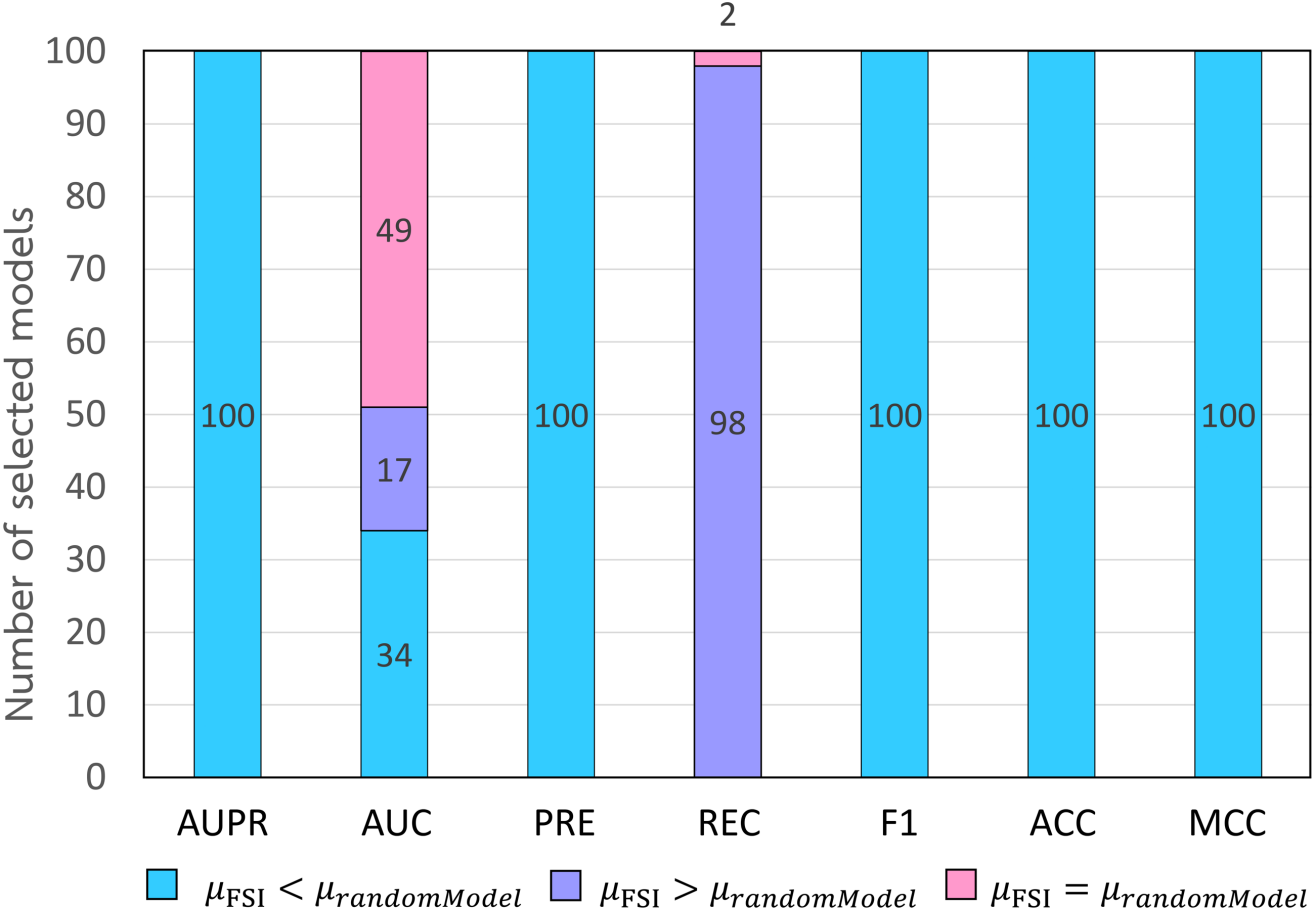
Performance comparison between 100 random integrating models and the FSI model.

In addition, we compared the performance of the FSI model with that of random similarity integrations. We randomly selected drugs and target similarities 100 times to integrate and construct 100 different models. Then, we compare the performance of the FSI model with that of 100 random integrating models. The results of the comparison are shown in Fig. 6.

**Figure 7:**
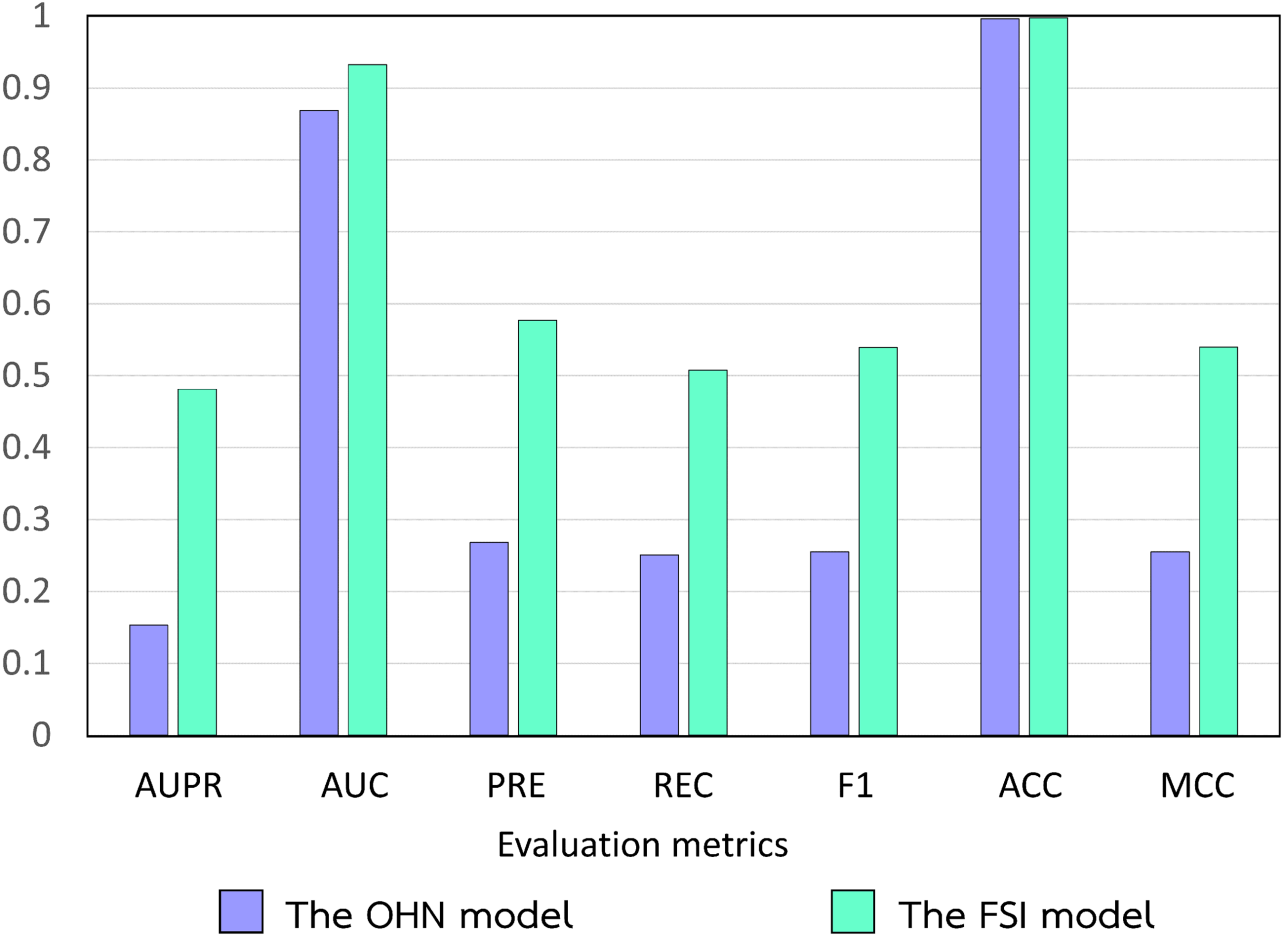
Performance comparison of the conventional model and the FSI model.

Based on each evaluation metric, Fig. 7 shows the results of the *t*-tests that compare the mean value of each evaluation metric of the FSI model with that of each random integrating model. In Fig. 6, the labeled numbers are the numbers of *t*-tests, resulting in the mean value of an evaluation metric of the FSI model being greater than (light blue bar) or lower than (purple bar) that of a random integrating model at a significance level of 0.05. The labeled numbers in the pink bar are the numbers of *t*-tests, resulting in the mean value of an evaluation metric of the FSI model not being different when compared to that of a random integrating model at a significance level of 0.05. The results showed that the mean values of all evaluation metrics of the FSI model, except AUC and REC, were significantly greater than those of the 100 random integrating models at a significance level of 0.05. This demonstrates that the model formed by systemically selecting drug and target similarities by FSI is more efficient than a model that randomly selects drug and target similarities without cautious consideration.

To predict DTIs, the network models utilize the drug–drug similarity derived only from drug chemical structures and the target–target similarity based only on local sequence alignments of target proteins. To show the superior performance of the model optimally integrating multiple drug and target similarities by FSI, we compared the performances of the OHN model (Liu et al., 2022; Liu et al., 2016; Peng et al., 2022; Wang, Yang & Li, 2013a) with those of the FSI model (Fig. 7). Using *t*-tests, we found that the FSI model performed significantly better than the OHN model at a significance level of 0.01, no matter what evaluation metrics were compared. The *t*-test *p*-values when considering AUPR, AUC, PRE, REC, F1, ACC, and MCC were 2.09E-11, 7.23E-08, 2.25E-09, 7.69E-11, 5.73E-11, 1.23E-06, and 5.53E-11, respectively. This means that integrating DDA_Jac and DDI_Cos into a drug–drug similarity derived from only drug chemical structures could greatly improve the performance of the OHN model in predicting DTIs.

### Significance of the drug similarity integration

In the FSI model, there were not only Structures and Seq_Loc used, but also two more similarities named DDA_Jac and DDI_Cos included. To verify the importance of these additional drug similarity measures for DTI predictions, we compared the performance of the FSI model with that of the removed DDA_Jac, namely the reduced DDA_Jac model, and removed DDI_Cos, namely the reduced DDI_Cos model. Moreover, we compared the performance of the FSI model with that of the models that permuted the DDA matrix, namely the permuted DDA model and the DDI matrix, namely the permuted DDI model. The mean AUPR, AUC, and F1 values of each model and the results of the *t*-tests are shown in Table 3.

**Table 3:** Performance comparison of the OHN model and the reduced model.

**Model**	**AUC**		**AUPR**		**F1**	
	**average**	***p*** **-value**	**average**	***p*** **-value**	**average**	***p*** **-value**
The FSI model	9.33E−01	–	4.81E−01	–	5.39E−01	–
The reduced DDA_Jac Model	9.22E−01^a^	6.46E−08	4.72E−01^a^	3.65E−06	5.31E−01^a^	3.91E−05
The reduced DDI_Cos Model	9.24E−01^a^	2.47E−07	4.71E−01^a^	1.43E−06	5.28E−01^a^	1.36E−06
Permuted DDA Model	9.32E−01^a^	9.9E−03	4.81E−01^b^	2.82E−02	5.38E−01^ns^	7.84E−02
Permuted DDI Model	9.14E−01^a^	5.19E−09	4.69E−01^a^	1.13E−06	5.24E−01^a^	1.03E−05
Permuted DDA and DDI Model	9.14E−01^a^	5.20E−09	4.69E−01^a^	1.11E−06	5.24E−01^a^	8.76E−06

**Notes.**

a Mean value of the FSI model is Significantly greater than those models at a significance level of 0.01.

b Mean value of the FSI model is Significantly greater than those models at a significance level of 0.05.

ns Not significance

Using *t*-tests, the mean values of AUC (0.4811), AUPR (0.4724), and F1 (0.4711) of the FSI models were greater than those of all the reduced models at a significance level of 0.01. This result showed that both DDA_Jac and DDI_Cos were important to DTI predictions, and we could not remove any of them from the heterogeneous network model. To verify the significance of the existing relationships in the DDA and DDI data, we generated the permuted models by randomly shuffling the existing edges in the DDA and DDI matrices, and then reconstructed heterogeneous network models with the permuted DDA_Jac and permuted DDI_Cos. Consequently, the mean AUC, AUPR, and F1 values of the FSI model were greater than those of the permuted DDI model and the model of both permuted DDAs and DDIs at a significance level of 0.01. Nevertheless, it cannot be concluded that the FSI model performs better than the permuted DDA model, especially when considering the mean F1 values. This may be due to the large sparsity of the DDA matrix, resulting in the fact that permuting this matrix does not change the DDA_Jac from the original one.

### Comparison with the random forest

To show the performance of our final model derived from the FSI algorithm to other methods, we employed a random forest with the features based on only the similarities used in this study. The results show that all accuracies of the heterogeneous network model were better than those of the random forest using the same sets of similarities as the features for training and testing, as shown in Table 4. Using the original similarities (which are Structure and Seq_Loc similarities), the random forest yielded better in term of AUC. Taking all similarities, the random forest and the heterogeneous network model showed similar AUC but different levels of accuracy where the heterogeneous network is much better. Furthermore, the heterogeneous network model with the obtained optimal similarities (from our FSI method) provided better performance than the random forest in terms of both accuracy and AUC. Obviously, the FSI algorithm provided an optimal set of similarities which improves the performance of the heterogeneous network model.

**Table 4:** Performance of heterogeneous network model and random forest.

	**Heterogeneous network model**		**Random Forest**	
	**Accuracy**	**AUC**	**Accuracy**	**AUC**
**Original similarities (OHN model)**	0.9650	0.8850	0.8322	0.9032
**All similarities (Full model)**	0.9780	0.9330	0.8737	0.9382
**Optimal similarities (FSI model)**	0.9980	0.9330	0.8533	0.9215

### Identification of new DTIs

In this section, the FSI model was used to predict new links between drugs and target proteins. We demonstrated the validation of the discovered DTIs through two types of case studies which were the case of target proteins with one known drug and the case of drugs with one known target protein. In the first case, we could obtain new drugs for a target protein with only one known drug. In the second case, we could predict new target proteins for a drug with only one known target. To find the list of new drugs for a certain target having only one known drug involved, we gathered the high-score predicted drug-target links. Three common targets which have about five proposed drugs were selected for more further verification and analysis (in Table 5). In the same manner, to find the list of new targets for a drug of interest having only one known target involved, in the high-score predicted drug-target links, common drugs having at least five proposed targets were selected as shown in Table 5. Three selected target proteins were tubulin beta-3 chain (Q13509), nicotinic acetylcholine receptor alpha-1 (P02708), and calcium/calmodulin-dependent 3′,5′-cyclic nucleotide phosphodiesterase 1C (Q14123), and two selected drugs were nicorandil (DB09220) and plerixafor (DB06809).

As shown in Table 5, the first row was the protein tubulin beta-3 chain (Q13509). Lxabepilone (DB04845) can bind to this protein and cause a reduction of improper cell division caused by several types of cancer, such as breast cancer, lung cancer, and lymphoma. From our predictions, the tubulin beta-3 chain could be associated with some drugs used in chemotherapy for cancer, including vincristine (DB00541), vinblastine (DB00570), and vinflunine (DB11641). Many studies have reported that these drugs interact with tubulins, such as for vincristine (Arora & Menezes, 2021; Lobert, Vulevic & Correia, 1996), for vinblastine (Arora & Menezes, 2021; Lobert et al., 1998), and for vinflunine (Aparicio, Pulido & Gallego, 2012; Arora & Menezes, 2021). Furthermore, beta-3 tubulin is predicted to be involved with albendazole (DB00518) and mebendazole (DB00643), which are drugs for helminth infections. It has been reported that both drugs interact with the alpha-1A and beta-4B tubulins (Berman et al., 2000; Chu et al., 2009; Ramírez et al., 2001; Solana et al., 2002). Thus, both albendazole and mebendazole could bind to a highly similar beta-3 tubulin protein.

**Table 5:** Target proteins and drugs from high-score predicted links with their possible drugs and targets, respectively.

**Targets**	**Known drug**	**Predicted drug** **(DrugBank ID)**
Tubulin beta-3 chain (Q13509)	Ixabepilone (DB04845)	DB00541^*^, DB00570^*^, DB11641^*^, DB00518, DB00643,
Nicotinic acetylcholine receptor alpha-1 (P02708)	Lamotrigine (DB00555)	DB00184^*^, DB00657^*^, DB01273, DB00514, DB00333
Calcium/calmodulin-dependent 3′,5′-cyclic nucleotide phosphodiesterase 1C (Q14123)	Caffeine (DB00201)	DB01023, DB01244, DB00622, DB01656, DB00651
**Drugs**	**Known Target**	**Predicted Target** **(Uniprot ID)**
Nicorandil (DB09220)	ATP-binding cassette sub-family C member 9 (O60706)	Q09428^*^, P13569, O15440, Q92887, P33527
Plerixafor (DB06809)	C-X-C chemokine receptor type 4 (P61073)	P35348, P08913, P35368, P18825, P18089

**Notes.**

* The drugs or targets that are found on publications are shown in bold numbers.

Next, the nicotinic acetylcholine receptor alpha-1 or nAChR *α*1 (P02708) is a protein that has been reported as a target protein for lamotrigine (DB00555). Lamotrigine is an antiepileptic drug approved for the treatment of epilepsy and bipolar disorder (Nishimura et al., 2010; Vallés, Garbus & Barrantes, 2007). When lamotrigine binds to nAChRs, voltage-dependent sodium channels on this protein are blocked and prevent the release of excitatory neurotransmitters, thereby preventing seizures (Prabhavalkar, Poovanpallil & Bhatt, 2015; Vallés, Garbus & Barrantes, 2007). According to the DTI predictions, nAChR *α*1 could be associated with drugs that play several roles in voltage-dependent ion channels of nAChR *α*1, including nicotine (DB00184) and mecamylamine (DB00657). Nicotine is a stimulant drug that acts as an agonist for nicotinic acetylcholine receptors (Wittenberg et al., 2020; Xiao et al., 2020). By binding this protein, nicotine activates voltage-gated calcium channels, causing the channel to open and allowing conductance of sodium, calcium, and potassium (Wishart et al., 2018). Nicotine is often used to relieve nicotine withdrawal symptoms and to aid in smoking cessation. Mecamylamine is a nicotine antagonist used to treat hypertension and uncomplicated malignant hypertension. By binding this protein, mecamylamine can act as a nicotinic acetylcholine receptor (nAChR) antagonist, inhibiting all known nAChR subtypes (Nickell et al., 2013). Many studies have shown that both drugs interact with nAChR *α*1, such as the studies of Cosford et al. (1996) and Holladay, Dart & Lynch (1997) for nicotine and the studies of Cosford et al. (1996) and Kaushal & Tadi (2020) for mecamylamine. Furthermore, nAChR *α*1 is predicted to involve varenicline (DB01273), dextromethorphan (DB00514), and methadone (DB00333), drugs for the relief of pain (Eyler, 2013; Hooten & Warner, 2015; Weinbroum et al., 2000) and treatment of addiction (Ali et al., 2017; Koyuncuoğlu & Saydam, 1990; Nocente et al., 2013). It has been revealed that these drugs interact with nAChR *α*4, nAChR *α*7, nAChR *α*4, and nACh *β*2 (Damaj et al., 2005; Lee et al., 2006; Mihalak, Carroll & Luetje, 2006; Steensland et al., 2007; Talka, Salminen & Tuominen, 2015; Talka, Tuominen & Salminen, 2015). Thus, varenicline, dextromethorphan, and methadone could bind to the highly similar protein nAChR *α*1.

Calcium/calmodulin-dependent 3′,5′-cyclic nucleotide phosphodiesterase 1C or PDE1C (Q14123) is another protein involving high-score predicted drug-target links. Caffeine (DB00201), a stimulant present in tea, coffee, and analgesic drugs, is the only approved known drug that interacts to target PDE1C. Caffeine can increase alertness in a short period of time. Caffeine can bind PDE1C and cause vasodilation (Echeverri et al., 2010). According to the DTI predictions, PDE1C could be associated with felodipine (DB01023), bepridil (DB01244), nicardipine (DB00622), and roflumilast (DB01656), which are drugs used in chemotherapy for cancer. Despite no clear evidence about these interactions, some publications support that these four drugs are involved in vasodilation, angina, and ischemic heart disease (Bansal & Khandelwal, 2019; DeWald et al., 2018; Ekins, Puhl & Davidow, 2020; Herych & Yatsyshyn, 2014). In addition, PDE1C is predicted to interact with dyphylline (DB00651), a drug approved for asthma, bronchospasm, and chronic obstructive pulmonary disease (COPD) (Ding et al., 2012; Wishart et al., 2018; Zhao et al., 2019). Interestingly, this drug and caffeine are in a class of methylxanthines, a purine-derived group of pharmacologic agents used for bronchodilation and stimulation (Basnet et al., 2017; Gottwalt & Tadi, 2021).

In the case of drugs having only one known targets, we selected nicorandil (DB09220) and plerixafor (DB06809) based on the high-score predicted drug-target links by the FSI model. Nicorandil is a vasodilatory drug used for patients with angina (Ahmed, 2019). It was found that the adenosine triphosphate (ATP)-binding cassette sub-family C member 9 or ABCC9 (O60706) interacted with nicorandil. This drug can activate vasodilation of arterioles and large coronary arteries (Rang et al., 2011; Wishart et al., 2018). According to the DTI predictions, nicorandil could be associated with some target proteins in the ABCC subfamily, including ATP-binding cassette subfamily C member 8 or ABCC8 (Q09428), cystic fibrosis transmembrane conductance regulator or ABCC7 (P13569), ATP-binding cassette subfamily C member 5 or ABCC5 (O15440), ATP-binding cassette subfamily C member 2 or ABCC2 (Q92887), and ATP-binding cassette sub-family C member 1 or ABCC1 (P33527). Interestingly, it was found that nicorandil can reduce an excess of insulin secretion in ABCC8-deficient insulin-producing cells (Guo et al., 2017). Moreover, some known ABCC9 drugs, such as adenosine triphosphate (DB00171) and glyburide (DB01016), have a link with predicted ABCCs proteins (Wishart et al., 2018). Because these proteins share the same known drugs as ABCC9, and they are also in the same subfamily, which could be rather structurally conserved, it is possible that other proteins in the ABCC subfamily may bind to nicorandil as well (Radivojac et al., 2013).

Plerixafor is an anti-HIV agent specifically active against T4-lymphotropic HIV strains (De Clercq, 2019). Plerixafor has been used to mobilize stem cells by blocking the interaction between *C* − *X* − *C* motif chemokine 12 or CXCL12 (P48061) and *C* − *X* − *C* chemokine receptor type 4 or CXCR4 (P61073) (Ilmer et al., 2020; Innis-Shelton & Costa, 2019; Wishart et al., 2018). CXCR4 is the only target protein that interacts with plerixafor. According to the DTI predictions, plerixafor could be associated with some target proteins in a group of adrenoceptors, including alpha-1A adrenergic receptor or ADRA1A (P35348), alpha-2A adrenergic receptor or ADRA2A (P08913), alpha-1B adrenergic receptor or ADRA1B (P35368), alpha-2C adrenergic receptor or ADRA2C (P18825), and alpha-2B adrenergic receptor or ADRA2B (P18089). Despite no clear evidence of the associations between plerixafor and these proteins, some proteins in the adrenoceptor group have been reported to induce mobilization of stem cells and progenitor cells (Agha et al., 2018; Asada et al., 2013; Baker et al., 2020; Dar et al., 2011).

## Discussion

To predict DTIs, the existing similarity-based methods (*e.g.*, Bleakley & Yamanishi, 2009; Campillos et al., 2008) mostly utilized the drug similarity only based on chemical structures and the target similarity only based on protein sequences. However, drug and target similarity measures solely based on a single feature of drugs and targets could introduce unreliable similarity scores and lead to inaccurate predictions of DTIs (Chen et al., 2021). In this study, we introduce a network propagation method with the FSI framework for systematically constructing an optimal model with suitable drug and target similarity integration. Through the FSI algorithm, we can select suitable drug and target similarity measures for similarity integration, the best integration method, and the best performance measure serving as the criteria for conducting the similarity integration.

As a result, the FSI model combines DDA_Jac, DDI_Cos, and Structures by using SNF as the integrated drug similarity and employs Sequences as the target–target similarity. By supplementing Structures with DDA_Jac and DDI_Cos, the FSI model perform significantly better than the OHN model, which utilizes only Structures and Sequences. This supports FSI efficiency in selecting and integrating beneficial information (*i.e.*, DDA_Jac and DDI_Cos) for predicting DTIs. Because the therapeutic effects of drugs are associated with the ability to modulate drug targets at the molecular level (Chen, Cheng & Li, 2020), the similarity based on drug indications could suggest relationships between drugs and targets. DDAs occur when two drugs involve related molecular targets or processes, resulting in unintended effects (Palleria et al., 2013). The high similarity based on DDIs could convey highly similar targets or processes that are involved in drugs. Several recent methods include DDAs and DDIs for more effective predictions of DTIs, such as those by Jiang et al. (2022) and Wang et al. (2022).

In addition to the drug and target similarity measures that are integrated, the performance of a model in predicting DTIs also depends on the similarity integration methods applied in the FSI algorithm. We studied both linear (*i.e.*, AVG, MAX, and MIN) and nonlinear (*i.e.*, SNF) functions for similarity integration. With a particular performance measure used in the FSI algorithm, we usually obtain different selected drug and target similarities when using different similarity integration methods. However, the integration method with the best performance in predicting DTIs is the SNF method (*i.e.*, Model 7 in Table 2). Linear integration is somewhat sensitive to outliers of some similarity scores. Rather than directly combining multiple similarity matrices, SNF is a network-based method that iteratively integrates multiple similarity networks into one composite network (Wang et al., 2014). Nowadays, SNF is an effective method widely exploited for aggregating multi-omics data in several biological applications, such as DTI prediction (Shao et al., 2022; Yan et al., 2017) and DDA inference (Jarada, Rokne & Alhajj, 2021a; Jarada, Rokne & Alhajj, 2021b).

In the FSI framework, the performance measure used for selecting drug and target similarity fusions is also important. We studied the most commonly used comprehensive metrics, including AUC, AUPR, and F1. According to the results (Table 1), using AUPR and F1 in FSI mostly obtains a similar selection of drug and target similarity measures. In addition, the models obtained using AUPR and F1 performed significantly better than those obtained using AUC (Table 2). This is because the number of negative (unknown) DTIs greatly outnumbers the number of positive (known) DTIs in the dataset, resulting in the AUC values being deceptive and unsuitable for evaluating model performance in this situation (Saito & Rehmsmeier, 2015).

In this work, the heterogeneous network model was improved by plugging in the feature selection and integration with our proposed FSI algorithm. The algorithm searched for a suitable integration method of various similarity measures and resulted in a set of similarities for the network propagation that yields better performance than the original one. The random forest which was performed with the same set of the features obtained by the FSI algorithm also yield better performance. In principle, the direct comparison of the heterogenous network model to the other state-of-the-art methods was not applicable since many current methods for drug-target predictions use various aspects of the features (not only similarities) to build up a prediction model and concern complicated tasks. Therefore, the prediction inference by the heterogeneous network model is simple and benefit for many applications and research studies. The development for improving this technique is still required and getting more interest as an essential tool now.

## Conclusions

In this study, we propose a network propagation method with the FSI framework, which systemically integrates multiple similarity measures, for predicting new links between drugs and targets. Based on our datasets, seven drug–drug similarity matrices and nine target–target similarity matrices can be constructed. Four different similarity integration methods and three comprehensive metrics were tested. Consequently, FSI selects to combine DDA_Jac, DDI_Cos, and Structures as a drug similarity by using AUPR and SNF. Additionally, the FSI model exploits Seq_Loc as a target similarity. The FSI model performed significantly better than the OHN model and the models with full and random similarity integration. This demonstrates the efficiency of the FSI framework in predicting DTIs. Nevertheless, the FSI framework is not limited to DTI prediction with our drug and target similarity measures. FSI can be generally applied for drug–target identification with different existing data sets, integration methods, and performance measures or even other applications requiring integration of multiple similarity measures.

##  Supplemental Information

10.7717/peerj-cs.1124/supp-1 Figure S1 Degree distributions in the drug–target bipartite network (A) degree distribution of drugs. (B) degree distribution of targets.

10.7717/peerj-cs.1124/supp-2 Table S1 The list of data used in this study and their sources

10.7717/peerj-cs.1124/supp-3 Table S2 Similarity measure methods and defined abbreviations of all drug data

10.7717/peerj-cs.1124/supp-4 Table S3 The similarity measures and defined abbreviations of all target protein data

## Additional Information and Declarations

### Data Availability

The following information was supplied regarding data availability: The data and code are available at GitHub: https://github.com/piyanuttnk/FSI_framework.
