# Supplementary material for: Heterogeneous network propagation with forward similarity integration to enhance drug–target association prediction
Source: PeerJ Comput Sci. 2022 Oct 11;8:e1124. doi: 10.7717/peerj-cs.1124 (PMC9575853; doi:10.7717/peerj-cs.1124)
Supplement: Figure S1 — (A) degree distribution of drugs. (B) degree distribution of targets. [file peerj-cs-08-1124-s001.pdf]

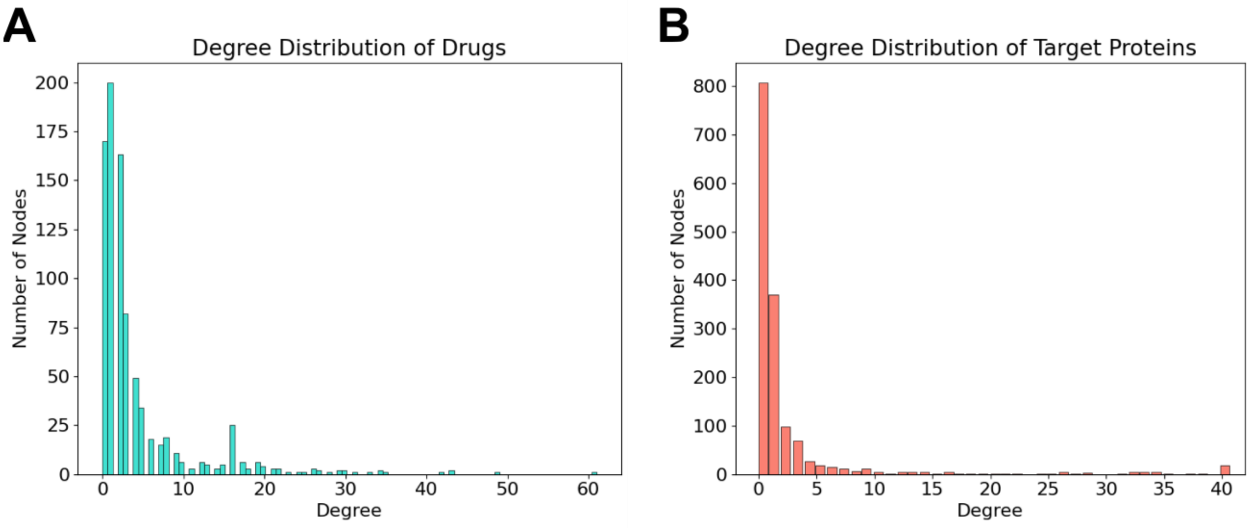

Supplemental Figure S1: Degree distributions in the drug–target bipartite network. (A) degree distribution of drugs. (B) degree distribution of targets.
