# Supplementary material for: Heterogeneous network propagation with forward similarity integration to enhance drug–target association prediction
Source: PeerJ Comput Sci. 2022 Oct 11;8:e1124. doi: 10.7717/peerj-cs.1124 (PMC9575853; doi:10.7717/peerj-cs.1124)
Supplement: Table S1 [file peerj-cs-08-1124-s002.docx]

**Supplemental Table S1: The list of data used in this study and their sources**

| Groups of data | Data sets | Data sources |
| --- | --- | --- |
| Drug-target interaction data | Drug-target interactions | DrugBank |
| Drug-related data | Chemical structures | DrugBank |
|  | Drug-drug interactions | DrugBank |
|  | Drug-disease associations | CTD |
|  | Drug side effects | SIDER |
| Target-related data | Protein sequences | DrugBank |
|  | Protein-protein interactions | STRING |
|  | GO annotations | GOA |
|  | Protein pathways | KEGG |
