# Supplementary material for: Heterogeneous network propagation with forward similarity integration to enhance drug–target association prediction
Source: PeerJ Comput Sci. 2022 Oct 11;8:e1124. doi: 10.7717/peerj-cs.1124 (PMC9575853; doi:10.7717/peerj-cs.1124)
Supplement: Table S2 [file peerj-cs-08-1124-s003.docx]

**Supplemental Table S2: Similarity measure methods and defined abbreviations of all drug data.**

| **Data of drugs** | **Similarity measure method** | **Defined**  **abbreviation** |
| --- | --- | --- |
| Chemical structures | Tanimoto coefficient | Structures |
| Drug-disease associations | Jaccard similarity | DDA_Jac |
|  | Cosine similarity | DDA_Cos |
| Drug-drug interactions | Jaccard similarity | DDI_Jac |
|  | Cosine similarity | DDI_Cos |
| Drug side effects | Jaccard similarity | SE_Jac |
|  | Cosine similarity | SE_Cos |
