# Supplementary material for: Heterogeneous network propagation with forward similarity integration to enhance drug–target association prediction
Source: PeerJ Comput Sci. 2022 Oct 11;8:e1124. doi: 10.7717/peerj-cs.1124 (PMC9575853; doi:10.7717/peerj-cs.1124)
Supplement: Table S3 [file peerj-cs-08-1124-s004.docx]

**Supplemental Table S3: The similarity measures and defined abbreviations of all target protein data.**

| **Data of target proteins** | **Similarity measures** | **Defined abbreviation** |
| --- | --- | --- |
| Protein sequences | Smith-Waterman algorithm | Seq_Loc |
|  | Needleman Wunsch algorithm | Seq_Glo |
| Protein-protein interactions | Inverse shortest path similarity | PPI_ISP |
|  | Jaccard similarity | PPI_Jac |
|  | Cosine similarity | PPI_Cos |
| GO annotations | GOSemsim: Wang method | GO_Wang |
|  | GOSemsim: Jiang Method | GO_Jiang |
| Protein pathways | Jaccard similarity | PW_Jac |
|  | Cosine similarity | PW_Cos |
